# Micro- and Nanocapsules Based on Artificial Peptides

**DOI:** 10.3390/molecules27041373

**Published:** 2022-02-17

**Authors:** Huayang Feng

**Affiliations:** Institute for Physical Chemistry, CeNIDE, University of Duisburg-Essen, 45141 Essen, Germany; huayang.feng@stud.uni-due.de

**Keywords:** capsules, artificial peptides, drug delivery

## Abstract

The encapsulation of active ingredients into solid capsules from biodegradable materials has received significant attention over the last decades. In this short review, we focus on the formation of micro- and nano-sized capsules and emulsions based on artificial peptides as a fully degradable material. It deals with various approaches for the preparation of peptide-based capsules as well as with their crucial properties such as size and stability. We categorize all preparation procedures into three basic approaches: self-assembly, polymerization and crosslinking, and layer-by-layer technology. This article is meant to offer a short overview over all successful methods suitable for obtaining access to these very promising carrier systems.

## 1. Introduction

Natural peptides, which are quite abundant in nature, are composed of amino acids linked by peptide bonds. Commonly, three different synthetic pathways are used to prepare peptides in the laboratory: by the polymerization of amino acids N-carboxyanhydrides (NCAs), amino acids N-thiocarboxyanhydrides (NTAs), and N-phenoxycarbonyl amino acids (NPCs); by various stepwise coupling reactions of α-amino acids; or by recombinant techniques for expressing peptides in microorganisms [1,2,3]. Different synthetic pathways tend to generate different kinds of peptides, such as oligopeptides and polypeptides. Various structures, such as nanotubes [4], nanofibers [5], hydrogels [6], nanovesicles [7], and nanocapsules [8], can be obtained through the self-assembly process of peptides. Moreover, because of their desirable properties such as versatile structure and great biocompatibility, these peptide-based architectures have been very promising in many fields, consisting of drug and gene delivery [9], photocatalysis [10], and photoelectric conversion [11]. Nevertheless, in this review, we mainly focus on peptide-based capsules.

The encapsulation of active ingredients (chemotherapeutic agents, antibiotic agents, mRNA, etc.) into various coating materials (lipids, polypeptides, albumin, chitosan, etc.) is intensely studied in the biomedical field for applications such as chemotherapy, oxygen carriers, COVID-19 vaccines, and so on [12,13]. Micro- and nanocapsules are colloidal particles that are formed by a shell-like wall with a liquid content according to a general and widely accepted classification of nanoparticles, while their homogeneously solid counterparts are often referred to as micro- and nanospheres [14]. Micro- and nanocapsules around hydrophobic cores can be prepared by four principally different approaches according to a previous review: interfacial polymerization, interfacial precipitation, interfacial deposition, and self-assembly procedures [14]. Considering the reports of peptide capsules in recent years, it is appropriate to classify the peptide-based capsules into three categories: capsules formed by self-assembly, by polymerization and crosslinking, and by layer-by-layer (LbL) technology. In the following, peptide-based capsules of these three different categories are introduced. Subsequently, essential properties of these capsules are discussed.

## 2. Current Methods for Peptide Micro/Nanocapsules Production

**Figure 1:**
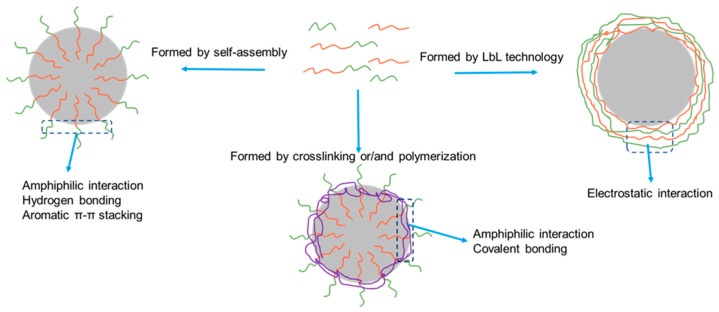
Schematic representation of methods used for peptide capsule preparation around hydrophobic cores: self-assembly (**left**); polymerization and cross-linking method (**below**); LbL technology (**right**).

The three basic approaches for the formation of peptide-based capsules, self-assembly, crosslinking, and LbL technology are depicted in Figure 1.

### 2.1. Capsule Formation by Self-Assembly

Through the self-assembly process, peptide-based capsules can be obtained via noncovalent interactions, including amphiphilic interactions, hydrogen bonding, and π-π stacking. In this case, the capsule wall is formed by self-assembly of amphiphilic peptides, a purely physical process without any chemical reaction (Figure 1). Some of the peptides may later form additional physical cross-linking through non-covalent bonding on the interface such as π-π stacking interactions or hydrogen bonding, weak interactions which further stabilize the outer membrane [15,16].

**Figure 2:**
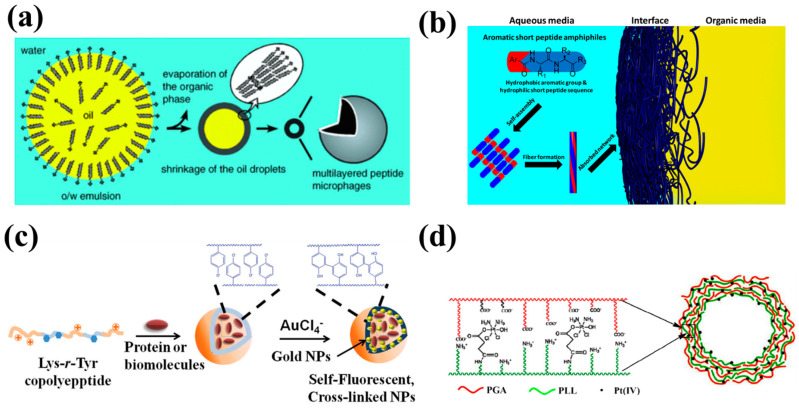
(**a**) Schematic illustration of the emulsion-templated self-assembly process [17]. (**b**) Cartoon of self-assembly and formation of fibrous network of aromatic short peptide amphiphiles at oil/water interface [21]. (**c**) Schematic illustration of biomolecule-loaded nanocapsules cross-linked by Au ion reduction [28]. (**d**) Schematic illustration of the structure of (PGA/PLL-Pt(IV))_3_ microcapsules [29].

Since capsules prepared from self-assembly around hydrophobic cores have no covalent interaction and have properties similar to coated droplets in an emulsion, the terms capsule dispersions and emulsions are equivalent at this point. In 2005, Morikawa et al. prepared hollow microcapsules based on α-helical peptide by the emulsion-templated self-assembly of amphiphilic poly(g-benzyl-L-glutamate) (Figure 2a). Corresponding hollow microcapsules are formed by rapid evaporation of the dichloromethane phase. The hollow microcapsules contain a solid polypeptide shell and an aqueous core, which is different from the normal capsules with a hydrophobic liquid core. The hollow microspheres are stable even after drying in air and can be used as carrier for both water-soluble and -insoluble molecules as reported [17].

Using an amphiphilic diblock-oligopeptide, nanocapsules around perfluorodecalin (PFD) were prepared by the self-assembly approach [18]. The capsule formation process leads to spherical shapes with an average diameter around 360 nm and a relatively narrow size distribution. With the PFD core, the capsules are capable of transporting oxygen and may be used as oxygen carriers for artificial blood replacement. The capsules exhibit fully reversible oxygen uptake capability comparable to other PFD systems [18].

In 2008, Hanson et al. prepared stable water-in-oil-in-water double emulsions and single oil-in-water emulsion using synthetic amphiphilic diblock copolypeptide surfactants of K_x_(rac-L)_y_ and K_x_L_y_ (K represents lysine and L represents leucine) [19]. Later, they prepared stable nano- and microscale emulsion droplets using block copolypeptide surfactants of biotin–K_55_(rac-L)_20_ and K_55_(rac-L)_20_, and they proved that the attachment of biotin to the hydrophilic segment shows no adverse effect on the stability of the emulsion [20].

In addition to the above-mentioned preparation of capsules through amphiphilic interactions, some researchers started to study emulsions specifically strengthened by hydrogen bonding and π-π stacking of short peptides. Compared to traditional emulsions, these emulsions stabilized by peptide show higher stability. In 2014, Ulijn et al. demonstrate the use of aromatic dipeptide to form the oil-in-water or water-in-oil emulsions that remain stable for months through the formation of nanofibrous networks at the organic/aqueous interface. Compared with traditional surfactant such as SDS, it is well justified to address these droplets as microcapsules, which considerably improve the stability of the dispersion (Figure 2b) [21].

Later, the same group began to study self-assembling of tripeptides in biphasic systems to stabilize microemulsions using a combined experimental/computational approach. They distinguished two types of aggregation behavior depending on peptide sequence: some of the tripeptides formed bilayer structures, and the others formed fiber-like morphologies. Significantly more stable emulsions are formed if the tripeptides have fibrillar assembly [22].

Yang et al. reported a considerable strategy to prepare highly stable nanoemulsions using ferrocene-tripeptide amphiphiles. The phase behavior regarding the emulsion–hydrogel transition and the size distribution of the emulsions could be precisely controlled by altering the temperature, the solvent ratio and the redox state of the ferrocene moiety [23].

Other interesting studies dealt with vesicles based on the self-assembly of peptides, the schematic drawing of which is shown elsewhere [24,25,26]. Considering that vesicles have analogue structures such as capsules, the related works based on self-assembly of peptides into vesicles are also briefly introduced at this point, even though no hydrophobic core is involved. In 2005, Holowka et al. reported the preparation of vesicles self-assembled from a series of poly(L-lysine)-b-poly(L-leucine) block copolypeptides as well as the poly(L-glutamatic acid)-b-poly(L-leucine) block copolypeptide. They proved that the proper ratio of diblock peptide chain length and the stable α-helical conformations in the oligoleucine segments are essential for the formation of the vesicles [24]. Hell et al. designed amphiphilic oligopeptides (Ac-Ala-Ala-Val-Val-Leu-Leu-Leu-Trp-Glu_2/7_-COOH), which were recombinantly produced in bacteria, that can self-assemble into vesicular structures. The vesicles formed from these amphiphilic peptides showed a radius of approximately 60 nm with a narrow particle size distribution. In addition, it was demonstrated that water-soluble molecules can be entrapped inside these peptide vesicles [27].

### 2.2. Capsule Formation by Polymerization and Crosslinking

In this case, the capsule wall is formed by polymerization and/or crosslinking of peptides at the interface between the solid and liquid phase of a dispersion or between the two liquid phases of an emulsion, a very efficient way to produce solid capsules owing to the stability of the covalent bonds (Figure 1).

Peptides consisting of cysteine and tyrosine are widely used in the preparation of capsules with cross-linked membranes [30,31]. Cysteine, as a natural cross-linking agent, serves as an important structural unit in many proteins thanks its capability to form disulfide bonds. Following this principle, peptide-based nanocapsules, which make use of cysteine as a cross-linking component, have been prepared. The capsules are based on an amphiphilic triblock oligopeptide consisting of blocks from aspartic acid, cysteine, and phenylalanine. The stability of the capsule wall was enhanced by the formation of sulfur bridges via the central cysteine block. The capsule formation process leads to spherical shapes with average diameters around 500 nm [Huayang Feng; Christian Mayer; et al. Interlayer-crosslinked capsules from synthetic triblock-peptides as potential artificial oxygen carriers. Angew. Chem., in preparation.]. The capsule walls show a distinct mechanical strength and allows for fast gas exchange.

Jacobs et al. reported a novel and facile way to prepare polypeptide nanospheres by miniemulsion polymerization of a S-(o-nitrobenzyl)-L-cysteine N-carboxyanhydrides (NCA) monomer. Subsequently, the polypeptide nanospheres were stabilized by forming disulfide bridges under the influence of UV light. The nanospheres show homogeneous size and diameters around 200 nm. This methodology provided a new way to prepare peptide-based nanoparticles [32].

Dityrosine exists in many biological macromolecules and is known as a natural cross-linker. Based on this, Huang et al. reported the fabrication of stable polypeptide-based nanocapsules consisting of L-lysine and L-tyrosine (Tyr) through cross-linking of Tyr residues by UV irradiation. They also used the cross-linked capsules for encapsulation of myoglobin. Their results indicate that the cross-linked membrane was permeable and that the function of the encapsulated protein would not be affected during the reduction or cross-linking reaction (Figure 2c) [28].

Min et al. reported the synthesis of short peptide nanocapsules with diameter of 100–200 nm through photo-polymerization of dityrosine in pH 10 buffer without requiring templates. The peptide nanocapsules showed high mechanical strength and stability. Interestingly, free-standing peptide thin films could be obtained by just changing reaction medium to methanol with 0.1 M NaOH [33].

Yang et al. prepared peptide-based nanospheres through self-assemble of ferrocene-tyrosine (Fc-Y) molecules, which could be transformed into hollow vesicles by covalent photo-crosslinking of the Fc-Y monomers. They proved that the peptide-based nanostructures have potential applications in various fields such as biomineralization of gold nanoparticles, biomimetic catalysis, and superior energy storage [34].

Apart from the above-mentioned preparation of capsules through crosslinking of tyrosine or cysteine, many peptide capsules based on other cross-linking strategies such as the amidation reaction have also been reported. In 2013, Wibowo et al. prepared stable cross-linked peptide-based capsules through the polymerization of a glutamate N-carboxyanhydrides (NCA) monomer on silica particle templates deposited by hyperbranched poly(ethylene imine) (PEI) macroinitiators, followed by deprotection, amidation reaction between PEI and Glu, and template dissolution [35]. Cavalieri et al. prepared polymeric capsules based on crosslinking of polylysine and PEG-NHS on the surface of mesoporous silica (MS) particles. After core removal procedure, siRNA could be loaded into the capsules. Their findings indicate that their peptide capsules have high siRNA loading capacity and could deliver siRNA to cells efficiently [36]. Wang et al. prepared polysaccharide-polypeptide hybrid spheres without crosslinking through interfacial polymerization of L-leucine NCA in O–W emulsion. Notably, amphiphilic polysaccharides play roles as macroinitiators and natural emulsifiers, which reduces the risk of potential side effects introduced by the emulsifier during the capsule formation process [37].

### 2.3. Capsule Formation by Layer-by-Layer (LbL) Technology

In this case, the capsule wall is formed by in-sequence adsorption of polyelectrolytes from the aqueous phase to the surface of solid particles or lipid droplets (Figure 1). If colloidal particles are used as templating inner cores, a procedure to dissolve the core material is also needed. After removal of the inner core, a hollow capsule is formed and can be used for the following encapsulation of a given active ingredient.

Cationic polylysine (PLL), polyornithine (POR), anionic polyglutamic acid (PGA), and polyaspartic acid (PAA) are four kinds of polypeptides that are commonly used for pairs of oppositely charged polyelectrolytes for formation of polypeptide multilayers [38,39]. Most of the corresponding peptide capsules are based on colloidal templates. Zhou et al. prepared multilayer microcapsules based on PGA and PLL for platinum-based pro-drug delivery with silica spheres as templates (Figure 2d) [29]. Huang et al. prepared peptide capsules based on PGA and POR. They extensively studied the physicochemical properties and biocompatibility of their capsules and found that capsules with positive charge and high stiffness facilitate a cellular uptake process [40]. Zhi et al. encapsulate an enzyme in polypeptide-based capsules with LbL deposition of PLL and PGA. Their results indicate that the polypeptide capsule wall can inhibit leakage of macromolecules from polypeptide-based capsules without precluding the permeation of small molecules [41]. Shutava et al. reported polypeptide nanoparticles based on PLL and PGA on gelatin nanoparticles by the LbL procedure, suitable for the delivery of natural polyphenols [42]. Modification with PEG and other biocompatible materials would improve the stability of these polypeptide capsules. Accordingly, Shutava et al. use PEG-modified PLL and PGA to prepare capsule walls using the LbL technology. Essentially, the modification with PEG preserves colloidal stability in the intermediate state when the surface charge of nanocapsules is low [43]. Ruttala et al. fabricated peptide capsules by the alternate deposition of polyarginine and PEG-b-PGA onto albumin conjugates. The encapsulation of drugs into the peptide capsules effectively prevented exposure of the drug to the systemic environment. Modification with PEG increased the blood circulation potential of the drugs, which is promising for clinical application [44]. Ye et al. reported the fabrication of robust and stable microcapsules with PLL-modified silk fibroin and graphene oxide flakes. The incorporation of graphene oxide significantly enhances the mechanical properties of the capsule, which can provide good protection for encapsulated cargo under harsh conditions [45].

In 2020, Mundo et al. prepared polypeptide stabilized soybean oil-in-water emulsions through LbL technology directly in the emulsions without using colloidal particles as template inner core. They initially prepared primary emulsions containing small anionic saponin-coated lipid droplets. Then, the primary emulsions were added to PLL solution and PGA solution in sequence to prepare secondary emulsions and the final tertiary emulsions [46]. Later, they also showed that multilayer emulsions could be formed by sequential adsorption of anionic emulsifier (Quillaja saponin), cationic polypeptide (PLL), and anionic polysaccharide layers onto the surfaces of lipid droplets [47].

Finally, LbL technology can be combined with the cross-linking strategy to produce more stable LbL capsules. Ye et al. fabricated microcapsules based on PLL and PGA-modified silk fibroin. They used the cross-linker 1-ethyl-3-[3-dimethylaminopropyl] carbodiimide hydrochloride (EDC) to promote the crosslinking of capsules. They focused on the physical (transport and mechanical) properties of the microcapsules after covalent cross-linking, and they demonstrated that the resulting capsules are a new promising platform for bioengineering applications [48]. Ochs et al. reported the synthesis of stable hollow capsules from PLL and PGA using a combination of click chemistry and LbL assembly, leading to capsules which are stable in the range of pH 2 to 11. Finally, LbL capsules can be easily modified with PEG and biotin to improve their physiological function [49].

## 3. Properties of Peptide Capsules

Size, mechanical stability, and dispersion stability are three of the key parameters that affect the performance of capsules as drug carriers. A summary of the different methods for peptide capsule synthesis and their basic properties is presented in Table 1.

In methods involving self-assembly, the properties of the oil–water interface, including surface tension and viscosity, can be used to adjust the size of the droplets, which determines the size of the capsules [50]. In the preparation methods, including ultrasonication, stirring, and microfluidics, parameters such as the ratio of hydrophobic solvent to water would have an effect on the size of capsules [51]. Generally, the higher the energy input to the system, the smaller the size of the capsules. Low fractions of the organic phase in relation to the aqueous phase also tend to yield smaller capsules. For LbL capsules, the size is mainly determined by the template core, and the capsule wall thickness is related to the number of layers. For polymerization and crosslinking, the capsule wall thicknesses are tunable by varying the polymerization time and initial monomer concentration. The composition of the capsules can also be controlled by using different amino acid N-carboxyanhydrides derivatives [35].

The mechanical stability of peptide-based capsules means the mechanical strength of the capsule wall, which mainly depends on the interactions among the peptides. Peptide capsules formed by crosslinking always show high mechanical stability, an effect that has been studied by many researchers [52]. It has been reported that the incorporation of graphene oxide could also enhance the mechanical properties of the capsule [45].

The dispersion stability refers to the ability of a dispersion to resist change in its properties over time. For capsules or emulsions formed only based on amphiphilic interactions, the dispersion stability can be adjusted by controlling the configuration and block ratio of the peptide. It has been proven that amphiphilic block copolypeptide containing a racemic (atactic) hydrophobic polypeptide block could stabilize emulsion droplets better than that with L-configuration (isotactic) hydrophobic polypeptide block, as racemic hydrophobic peptides such as poly(rac-leucine) are soluble in many organic solvents such as CH_2_Cl_2_ and (CH_3_)_2_SO, whereas poly(L-leucine) is not. Amphiphilic block copolypeptides with longer hydrophilic chains are especially suited for the stabilization of oil-in-water emulsions where the oil is on the concave side of the curved interface of a nanoscale droplet. Conversely, the inner water–oil interface of a WOW double emulsion is best stabilized by an amphiphilic block copolypeptide with shorter hydrophilic chains because the oil is on the convex side of the interface [19]. Although peptides with amphiphilic properties would accumulate at the oil–water interface and stabilize the emulsion, the oil phase would merge or precipitate over time due to the fact that oil and water notoriously do not mix. The formation of capsules through π-π stacking interactions and hydrogen bonding of amphiphilic short peptides significantly improves the dispersion stability of the emulsions owing to the formation of nanofibrous networks at the organic/aqueous interface [21]. Modifications with PEG and other biocompatible materials would not only stabilize the capsules but also extend the in vivo circulation time [53]. Various types of functionalities, such as the tendency to penetrate into cells, would also be achieved by modifications with specific functional materials [54].

## 4. Conclusions and Perspectives

Peptides have attracted researchers’ interest because of their versatile composition, attainable secondary structures, attractive self-assembly behavior, and ideal biocompatibility and biodegradability. It is now possible to prepare peptide-based capsules with defined sizes and functionalities through a variety of methods. Based on these developments, peptide-based capsules have been utilized in various types of bio-applications, including drug and gene delivery. This review mainly focuses on the preparation of peptide-based capsules and emulsions through different approaches. Furthermore, the combination of various cross-linking strategies with self-assembly and LbL methods is undoubtedly a promising pathway for preparing capsules with enhanced mechanical stability. A more flexible design of biomedical capsules could combine peptides with other biocompatible materials, leading to specific functionalities and high mechanical stability. Overall, considering the progress in developing peptide-based capsules and emulsions, we believe that peptide-based capsules will have a very promising potential for biomedical applications.

## Figures and Tables

**Table 1:** Summary of different formation processes of peptide capsules and the resulting capsule properties.

Formation Process	Main Interaction	Peptide	Mechanical Stability	Diameter	Ref.
Self-assembly	Amphiphilic interaction and hydrogen bonding	Polypeptide	Average	1–5 μm	[17]
Self-assembly	Amphiphilic interaction	Polypeptide	Average	100 nm−1 μm	[18]
Self-assembly	Amphiphilic interaction	Polypeptide	Average	10–100 nm	[19]
Self-assembly	Aromatic π-π stacking and hydrogen bonding	Dipeptide	High	5–50 μm	[21]
Self-assembly	Aromatic π-π stacking and hydrogen bonding	Tripeptide	High	1–10 μm	[22]
Self-assembly	Amphiphilic interaction and hydrogen bonding	Polypeptide	Average	1–10 μm	[24]
Self-assembly	Amphiphilic interaction and hydrogen bonding	Oligopeptides	Average	100–200 nm	[27]
Crosslinking	Covalent bonding	Polypeptide	High	100–500 nm	[32]
Crosslinking	Covalent bonding	Polypeptide	High	20–200 nm	[28]
Crosslinking	Covalent bonding	Oligopeptides	High	100–200 nm	[33]
Crosslinking	Covalent bonding	Polypeptide	High	5 μm	[35]
Crosslinking	Covalent bonding	Polypeptide	High	1–2 μm	[36]
LbL technology	Electrostatic interaction	Polypeptide	Average	500 nm−2 μm	[29]
LbL technology	Electrostatic interaction	Polypeptide	Average	1–5 μm	[40]
LbL technology	Electrostatic interaction	Polypeptide	Average	4–5 μm	[41]
LbL technology	Electrostatic interaction	Polypeptide	Average	100–200 nm	[44]
LbL technology	Electrostatic interaction	Polypeptide	Average	500 nm–2 μm	[46]
LbL technology	Electrostatic interaction and covalent bonding	Polypeptide	High	3–6 μm	[48]
LbL technology	Electrostatic interaction and covalent bonding	Polypeptide	High	5–10 μm	[49]

## References

[1] Rasines Mazo A., Allison-Logan S., Karimi F., Chan N.J., Qiu W., Duan W., O’Brien-Simpson N.M., Qiao G.G. (2020). Ring opening polymerization of alpha-amino acids: Advances in synthesis, architecture and applications of polypeptides and their hybrids. Chem. Soc. Rev..

[2] Endo T., Sudo A. (2020). Well-defined construction of functional macromolecular architectures based on polymerization of amino acid urethanes. Biomedicines.

[3] Zheng B., Bai T., Tao X., Ling J. (2021). An inspection into multifarious ways to synthesize poly(amino acid)s. Macromol. Rapid Commun..

[4] Hamley I.W. (2014). Peptide nanotubes. Angew. Chem. Int. Ed..

[5] Arslan E., Garip I.C., Gulseren G., Tekinay A.B., Guler M.O. (2014). Bioactive supramolecular peptide nanofibers for regenerative medicine. Adv. Healthc. Mater..

[6] Li J., Xing R., Bai S., Yan X. (2019). Recent advances of self-assembling peptide-based hydrogels for biomedical applications. Soft Matter.

[7] Fatouros D.G., Lamprou D.A., Urquhart A.J., Yannopoulos S.N., Vizirianakis I.S., Zhang S., Koutsopoulos S. (2014). Lipid-like self-assembling peptide nanovesicles for drug delivery. ACS Appl. Mater. Interfaces.

[8] Matsuura K., Watanabe K., Matsushita Y., Kimizuka N. (2013). Guest-binding behavior of peptide nanocapsules self-assembled from viral peptide fragments. Polym. J..

[9] He C., Zhuang X., Tang Z., Tian H., Chen X. (2012). Stimuli-sensitive synthetic polypeptide-based materials for drug and gene delivery. Adv. Healthc. Mater..

[10] Liu K., Xing R., Chen C., Shen G., Yan L., Zou Q., Ma G., Mohwald H., Yan X. (2015). Peptide-induced hierarchical long-range order and photocatalytic activity of porphyrin assemblies. Angew. Chem. Int. Ed. Engl..

[11] Wang S., Zhang D., Zhang X., Yu D., Jiang X., Wang Z., Cao M., Xia Y., Liu H. (2019). Short peptide-regulated aggregation of porphyrins for photoelectric conversion. Sustain. Energy Fuels.

[12] Hou X., Zaks T., Langer R., Dong Y. (2021). Lipid nanoparticles for mRNA delivery. Nat. Rev. Mater..

[13] Martinez Rivas C.J., Tarhini M., Badri W., Miladi K., Greige-Gerges H., Nazari Q.A., Galindo Rodriguez S.A., Roman R.A., Fessi H., Elaissari A. (2017). Nanoprecipitation process: From encapsulation to drug delivery. Int. J. Pharm..

[14] Mayer C. (2005). Nanocapsules as drug delivery systems. Int. J. Artif. Organs.

[15] Wang J., Liu K., Xing R., Yan X. (2016). Peptide self-assembly: Thermodynamics and kinetics. Chem. Soc. Rev..

[16] Fleming S., Ulijn R.V. (2014). Design of nanostructures based on aromatic peptide amphiphiles. Chem. Soc. Rev..

[17] Morikawa M.A., Yoshihara M., Endo T., Kimizuka N. (2005). alpha-Helical polypeptide microcapsules formed by emulsion-templated self-assembly. Chemistry.

[18] Feng H., Linders J., Myszkowska S., Mayer C. (2021). Capsules from synthetic diblock-peptides as potential artificial oxygen carriers. J. Microencapsul..

[19] Hanson J.A., Chang C.B., Graves S.M., Li Z., Mason T.G., Deming T.J. (2008). Nanoscale double emulsions stabilized by single-component block copolypeptides. Nature.

[20] Hanson J.A., Deming T.J. (2011). Functionalized nanoscale through microscale polypeptide stabilized emulsions for display of biomolecules. Polym. Chem..

[21] Bai S., Pappas C., Debnath S., Frederix P.J.M., Leckie J., Fleming S., Ulijn R.V. (2014). Stable emulsions formed by self-assembly of interfacial networks of dipeptide derivatives. ACS Nano.

[22] Scott G.G., McKnight P.J., Tuttle T., Ulijn R.V. (2016). Tripeptide emulsifiers. Adv. Mater..

[23] Yang X., Wang Y., Qi W., Su R., He Z. (2017). Bioorganometallic ferrocene-tripeptide nanoemulsions. Nanoscale.

[24] Holowka E.P., Pochan. D.J., Deming A.T.J. (2005). Charged polypeptide vesicles with controllable diam. JACS.

[25] Hamley I.W. (2011). Self-assembly of amphiphilic peptides. Soft Matter.

[26] Bellomo E.G., Wyrsta M.D., Pakstis L., Pochan D.J., Deming T.J. (2004). Stimuli-responsive polypeptide vesicles by conformation-specific assembly. Nat. Mater..

[27] Hell A.J.v., Costa C.I.C.A., Flesch F.M., Sutter M., Jiskoot W., Crommelin D.J.A., Hennink W.E., Mastrobatti E. (2007). Self-Assembly of recombinant amphiphilic oligopeptides into vesicles. Biomacromolecules.

[28] Huang Y.F., Lu S.C., Huang Y.C., Jan J.S. (2014). Cross-linked, self-fluorescent gold nanoparticle/polypeptide nanocapsules comprising dityrosine for protein encapsulation and label-free imaging. Small.

[29] Zhou D., Xiao H., Meng F., Zhou S., Guo J., Li X., Jing X., Huang Y. (2012). Layer-by-layer assembled polypeptide capsules for platinum-based pro-drug delivery. Bioconjug. Chem..

[30] Wong M.S., Cha J.N., Choi K.-S., Deming T.J., Stucky G.D. (2002). Assembly of nanoparticles into hollow spheres using blcok copolypeptides. Nano Lett..

[31] Lee J., Ju M., Cho O.H., Kim Y., Nam K.T. (2019). Tyrosine-rich peptides as a platform for assembly and material synthesis. Adv. Sci..

[32] Jacobs J., Pavlovic D., Prydderch H., Moradi M.A., Ibarboure E., Heuts J.P.A., Lecommandoux S., Heise A. (2019). Polypeptide nanoparticles obtained from emulsion polymerization of amino acid N-carboxyanhydrides. J. Am. Chem. Soc..

[33] Min K.I., Yun G., Jang Y., Kim K.R., Ko Y.H., Jang H.S., Lee Y.S., Kim K., Kim D.P. (2016). Covalent self-assembly and one-step photocrosslinking of tyrosine-rich oligopeptides to form diverse nanostructures. Angew. Chem. Int. Ed. Engl..

[34] Yang X., Wang Y., Qi W., Zhang J., Zhang L., Huang R., Su R., He Z. (2018). Photo-induced polymerization and reconfigurable assembly of multifunctional ferrocene-tyrosine. Small.

[35] Wibowo S.H., Wong E.H., Sulistio A., Guntari S.N., Blencowe A., Caruso F., Qiao G.G. (2013). Assembly of free-standing polypeptide films via the synergistic combination of hyperbranched macroinitiators, the grafting-from approach, and cross-chain termination. Adv. Mater..

[36] Cavalieri F., Beretta G.L., Cui J., Braunger J.A., Yan Y., Richardson J.J., Tinelli S., Folini M., Zaffaroni N., Caruso F. (2015). Redox-sensitive PEG-polypeptide nanoporous particles for survivin Ssilencing in prostate cancer cells. Biomacromolecules.

[37] Wang J., Liu C., Chi P. (2009). One-step preparation of glycopeptide microspheres based on alpha-amino acid-N-carboxyanhydride polymerization using interfacial protocols. J. Biomed. Mater. Res. B Appl. Biomater..

[38] Xiao F.X., Pagliaro M., Xu Y.J., Liu B. (2016). Layer-by-layer assembly of versatile nanoarchitectures with diverse dimensionality: A new perspective for rational construction of multilayer assemblies. Chem. Soc. Rev..

[39] Borges J., Mano J.F. (2014). Molecular interactions driving the layer-by-layer assembly of multilayers. Chem. Rev..

[40] Huang W., Zhang T., Shi P., Yang D., Luo S., Voit B., Appelhans D., Zan X., Chen H. (2019). The construction and effect of physical properties on intracellular drug delivery of poly(amino acid) capsules. Colloids Surf. B Biointerfaces.

[41] Zhi Z.L., Haynie D.T. (2006). Straightforward and effective protein encapsulation in polypeptide-based artificial cells. Artif. Cells Blood Substit. Biotechnol..

[42] Shutava. T.G., Balkundi. S.S., Vangala. P., Steffan. J.J., Bigelow. R.L., Cardelli. J.A., O’Neal. D.P., Yuri M.L. (2009). Layer-by-layer-coated gelatin nanoparticles as a vehicle for delivery of natural polyphenols. ACS Nano.

[43] Shutava T.G., Pattekari P.P., Arapov K.A., Torchilin V.P., Lvov Y.M. (2012). Architectural layer-by-layer assembly of drug nanocapsules with PEGylated polyelectrolytes. Soft Matter.

[44] Ruttala H.B., Ramasamy T., Shin B.S., Choi H.G., Yong C.S., Kim J.O. (2017). Layer-by-layer assembly of hierarchical nanoarchitectures to enhance the systemic performance of nanoparticle albumin-bound paclitaxel. Int. J. Pharm..

[45] Ye C., Combs Z.A., Calabrese R., Dai H., Kaplan D.L., Tsukruk V.V. (2014). Robust microcapsules with controlled permeability from silk fibroin reinforced with graphene oxide. Small.

[46] Muriel Mundo J.L., Zhou H., Tan Y., Liu J., McClements D.J. (2020). Stabilization of soybean oil-in-water emulsions using polypeptide multilayers: Cationic polylysine and anionic polyglutamic acid. Food Res. Int..

[47] Mundo J.L.M., Zhou H., Tan Y., Liu J., McClements D.J. (2021). Enhancing emulsion functionality using multilayer technology: Coating lipid droplets with saponin-polypeptide-polysaccharide layers by electrostatic deposition. Food Res. Int..

[48] Ye C., Drachuk I., Calabrese R., Dai H., Kaplan D.L., Tsukruk V.V. (2012). Permeability and micromechanical properties of silk ionomer microcapsules. Langmuir.

[49] Ochs C.J., Such G.K., Städler B., Caruso F. (2008). Low-fouling, biofunctionalized, and biodegradable click capsules. Biomacromolecules.

[50] McClements D.J., Gumus C.E. (2016). Natural emulsifiers—Biosurfactants, phospholipids, biopolymers, and colloidal particles: Molecular and physicochemical basis of functional performance. Adv. Colloid. Interface Sci..

[51] Shimanovich U., Bernardes G.J., Knowles T.P., Cavaco-Paulo A. (2014). Protein micro- and nano-capsules for biomedical applications. Chem. Soc. Rev..

[52] Wibowo S.H., Sulistio A., Wong E.H., Blencowe A., Qiao G.G. (2014). Polypeptide films via N-carboxyanhydride ring-opening polymerization (NCA-ROP): Past, present and future. Chem. Commun..

[53] Lv S., Tang Z., Li M., Lin J., Song W., Liu H., Huang Y., Zhang Y., Chen X. (2014). Co-delivery of doxorubicin and paclitaxel by PEG-polypeptide nanovehicle for the treatment of non-small cell lung cancer. Biomaterials.

[54] Holowka E.P., Sun V.Z., Kamei D.T., Deming T.J. (2007). Polyarginine segments in block copolypeptides drive both vesicular assembly and intracellular delivery. Nat. Mater..

